# Medical News

**Published:** 1886-02

**Authors:** 


### Medical News.

Dr. Albert Smith, the distinguished gynæcologist, died at his home in Philadelphia, Dec. 14, 1885.

The recent small-pox epidemic in Montreal will cost, according to the City Auditor, about $100,000.

A Spanish physician has discovered bacilli, which look exactly like the comma bacilli of Koch, in various specimens of tincture of opium.

President Cleveland was elected an honorary member of the American Public Health Association at the recent annual meeting. He is the first honorary member ever elected by this body.

A New York judge has lately decided that a landlord is responsible for the sanitary condition of the house he rents. In this case, tenants refused to pay the rent of the house they occupied, because it contained sewer gas, and from which they had removed before the expiration of their lease. The landlord sued for the amount due him on the remainder of the lease. Tenants gained the suit.

The "Congress Controversy" is attracting considerable attention in the French journals. *Le Journal de Med. de Paris*, for November 1st, speaks of the profession in America as being in the "prettiest state of discord imaginable." "The New Code Party, who favor consultations with homœopaths, herb doctors, and other respectable quacks *ejusdem farinæ*," are looked upon as the source of all the trouble.

Drs. Vibert and Ogier have recently made the discovery that albumen is present in the urine of cadavers in eighty per cent. of the bodies examined. They conclude that the albumen is produced by the decomposition of vesical mucus. This discovery is significant, since death has been attributed to albuminuria in cases where no other cause could be discovered, simply because a chemical test demonstrated the presence of albumen in the urine.

